# Transition Metal Hollow Nanocages as Promising Cathodes for the Long-Term Cyclability of Li–O_2_ Batteries

**DOI:** 10.3390/nano8050308

**Published:** 2018-05-07

**Authors:** Amrita Chatterjee, Siu Wing Or, Yulin Cao

**Affiliations:** 1 Department of Electrical Engineering, The Hong Kong Polytechnic University, Hung Hom, Kowloon, Hong Kong; amrita.chatterjee@polyu.edu.hk (A.C.); caoyulin@szpt.edu.cn (Y.C.); 2 Physics Laboratory, Industrial Training Center, Shenzhen Polytechnic, Shenzhen 518055, China

**Keywords:** electrocatalytic cathodes, hollow nanocages, Li–O_2_ batteries, cyclic stability, transition metals

## Abstract

As a step towards efficient and cost-effective electrocatalytic cathodes for Li–O_2_ batteries, highly porous hausmannite-type Mn_3_O_4_ hollow nanocages (MOHNs) of a large diameter of ~250 nm and a high surface area of 90.65 m^2^·g^−1^ were synthesized and their physicochemical and electrochemical properties were studied in addition to their formation mechanism. A facile approach using carbon spheres as the template and MnCl_2_ as the precursor was adopted to suit the purpose. The MOHNs/Ketjenblack cathode-based Li–O_2_ battery demonstrated an improved cyclability of 50 discharge–charge cycles at a specific current of 400 mA·g^−1^ and a specific capacity of 600 mAh·g^−1^. In contrast, the Ketjenblack cathode-based one can sustain only 15 cycles under the same electrolytic system comprised of 1 M LiTFSI/TEGDME. It is surmised that the unique hollow nanocage morphology of MOHNs is responsible for the high electrochemical performance. The hollow nanocages were a result of the aggregation of crystalline nanoparticles of 25–35 nm size, and the mesoscopic pores between the nanoparticles gave rise to a loosely mesoporous structure for accommodating the volume change in the MOHNs/Ketjenblack cathode during electrocatalytic reactions. The improved cyclic stability is mainly due to the faster mass transport of the O_2_ through the mesoscopic pores. This work is comparable to the state-of-the-art experimentations on cathodes for Li–O_2_ batteries that focus on the use of non-precious transition materials.

## 1. Introduction

In this age of global warming, it is considerably difficult to meet the high energy demand of ~14 TW by 2050 [1]. However, the recent achievements of next-generation Li−air or Li–O_2_ batteries show promise towards realizing this dream in a sustainable way. A typical Li−O_2_ battery is composed of a Li anode and an O_2_ cathode and it combines the advantages of fuel cells and batteries. The chemical energy is converted to electrical energy during discharge, while the electric energy is stored by splitting the discharged products during charging. The specific energy of Li−O_2_ batteries is ~800 Wh/kg which is about four times that of current Li-ion batteries. Modeling estimations show that the theoretical energy density of nonaqueous Li−O_2_ batteries is 3436 Wh·L^−1^ in contrast to 2234 Wh·L^−1^ in aqueous Li−O_2_ batteries. Hence, ether and carbonate-based electrolytes have garnered more attention [2]. In spite of the potential advantages of Li–O_2_ batteries, their realization has suffered obstacles, primarily due to the lack of a suitable cathode that can prevent the formation of the discharge product––lithium peroxide (Li_2_O_2_) during the oxygen evolution reaction (OER). The toroidal and non-conductive Li_2_O_2_ compounds formed during the electrochemical reactions tend to block the pores or passivate of the active sites of the cathode. Ideally, a mesoporous structure with a high concentration of exposed active sites is suitable as a cathode. The mesoporosity of the catalyst can reduce the pore blockage by undesirable side products [3]. 

The current status of the Li−air or Li−O_2_ batteries is still a far reach in comparison to the state-of-the-art Li-ion batteries. While, Li-ion batteries and PEM fuel cells can be discharged−charged at a current density of 10 and 1500 mA·cm^2^, respectively, Li−O_2_ batteries can only sustain 0.1−0.5 mA·cm^2^ of current density. This results in a <60% voltage efficiency for Li−O_2_ batteries in contrast to the 90% voltage efficiency achieved in Li-ion batteries. The huge discrepancies in the theoretical and practical data are due to the lack of a properly designed cathode for Li−O_2_ batteries. In absence of a suitable cathode, the kinetics of electrocatalytic reactions of ORR–OER (oxygen reduction reaction–oxygen evolution reaction) are sluggish. This causes the formation of LiO_2_ which further dis-proportionate to insulating Li_2_O_2_ [4,5,6].

To mitigate these challenges, various electrocatalysts have been experimented on as cathodes for Li−O_2_ batteries. Some of them are porous carbon materials, including carbon black, nanostructured/functionalized carbon, nanostructured diamond-like carbon, graphene, transition metal oxides, especially manganese and cobalt-based metals, non-precious metals, and precious metals such as platinum, palladium, and gold. Among these, transition metal oxides have garnered attention due to their cost-effectiveness, easy availability, their facile method of synthesis, high electrochemical performance, stability, and controlled tunable morphology [4,7].

Oxides of Co, Mn, Ni, and Ru are popularly investigated for their catalytic activity. Bruce et al. have been the pioneers to show that α-MnO_2_ possesses superior electrochemical properties for ORR*–*OER. Since then, various crystalline structures of MnO_2_, MnO, MnOOH, Mn_2_O_3_, and Mn_3_O_4_ have been investigated for ORR−OER [8,9,10]. The best performances of MnO_2_ with above 100 cycles of stability at a >500 mAh·g^−1^ discharge voltage involves their composites with carbon nanotubes, graphene, or noble metals [11]. In this regard, the spinel-type transition metal oxides are favored as cathodes for Li−O_2_ batteries due to the easy control of their shape and size [12]. The electrochemical characteristics of spinel shape structures arise because they can accommodate a wide range of multiple oxidation states cations in their tetrahedral and octahedral sites. Such atomic configurations and coordination dictate the catalytic ORR−OER [13]. Spinel-type transition metal oxides of Mn, Co, and Ni have been reported as potential cathodes for Li−O_2_ batteries. Investigations on Co_3_O_4_, NiCo_2_O_4_, CoFe_2_O_4_, CoMn_2_O_4_, and MnCo_2_O_4_ have concluded that the oxygen binding ability of the defect sites on the metal oxides and their redox capability determine the catalytic activity of the materials. Morphologies such as spinel nanofibers or hollow porous structure facilitate the diffusion of reactants and reduce the voltage gaps [12].

Though there are reports investigating the structure-activity relationships of Mn_3_O_4_ as electrodes for Li-ion batteries and electrocatalysts for ORR−OER [14,15], there are very few results focusing on the applications of Mn_3_O_4_ as cathodes for Li−O_2_ batteries [16]. Wu et al. have developed a composite of Mn_3_O_4_ with reduced graphene oxide that showed stability up to 20 cycles at 500 mA·g^−1^. It has recorded the highest initial discharge capacity of 16,200 mAh·g^−1^, which surpasses the state-of-the-art Pt/C catalyst [17]. The bifunctional 1-D Mn_3_O_4_/carbon nanofiber composite developed by Jung et al. reduces the overpotential to 0.08 V during ORR−OER. However, it is also reported that with a higher number of cycles, the charge voltage increases due to the oxidation of the carbon support [18]. Therefore, it is necessary to tune the morphology of Mn_3_O_4_ to reduce the use of expensive and oxidation prone carbon supports as an approach towards cost-effective catalysts. 

One of the drawbacks of using these spinel structured oxides is their low surface area [12]. In this work, we have demonstrated that template assisted growth of spinel-type manganese oxide nanocages (MOHNs) can lead to a high surface area of 90.65 m^2^·g^−1^ of these porous materials. The particles of 25−35 nm diameter agglomerate to form a loose mesoporous hollow nanocage structure. The MOHNs/Ketjenblack cathode shows improved catalytic effect in ORR−OER due to its mesoporous structure. The hollow nanocage structure provides a pathway for better diffusion of reactants and products as well as prevents the pore blockage by the undesired Li_2_O_2_. Therefore, the active sites of the MOHNs are not passivated rapidly and improve the cyclic stability the Li–O_2_ batteries to sustain at least 50 cycles. 

## 2. Materials and Methods 

### 2.1. Materials

The reagents used were glucose, manganese chloride tetrahydrate, Li foil, Whatman GF/D glass fiber, Ketjenblack (KB), polytetrafluoroethylene (PTFE), 1 M of LiTFSI/TEGDME (lithium *bis*(trifluoromethanesulfonyl)imide/(tetra(ethylene glycol)dimethyl ether)), absolute ethanol, and carbon paper discs (TGP-H-060). All reagents were obtained from Sigma-Aldrich (Hong Kong) and used without further purification unless otherwise stated. 

### 2.2. Synthesis of Mn_3_O_4_ Hollow Nanocages (MOHNs)

The MOHNs were prepared from a hard carbon template. Typically, 8 g of glucose was dissolved in 40 mL of deionized water to form a clear solution. The solution was heated in a Teflon-lined 50 mL autoclave at 160 °C for 22 h. The product obtained was centrifuged, washed and re-dispersed in water several times. The process was repeated with ethanol. Finally, the isolated carbon nanospheres were dried in an oven at 80 °C for 12 h [19]. 

To prepare the MOHNs from the carbon nanosphere template, 4 g of the template was re-dispersed in 50 mL of 0.5 M MnCl_2_ solution. The slurry was ultrasonicated for 50 min and aged for 12 h at room temperature. The product was isolated and washed with deionized water and heated at 450 °C for 1 h in the presence of air. Finally, the brown powders of MOHNs were obtained. 

### 2.3. Physicochemical Characterizations of MOHNs

The crystalline structure of the oxygen-electrode materials was determined using the X-ray diffractometer (Bruker D8 advance, Karlsruhe, Germany) with Ni-filtered CuKα line as a radiation source. To complement the microstructure information obtained from X-Ray Diffraction patterns, Raman spectroscopy was performed. The Raman spectra were taken at room temperature using Raman spectroscopy 2000 (Renishaw, UK) equipped with a CCD camera and an optical microscope which provided the laser beam. A red line (633 nm) was taken as a back-scattering source. The surface area and porosity of the developed cathode materials were studied from the nitrogen physisorption isotherms at 77 K using Nova Station A (Quantachrome Instruments, Boynton Beach, FL, USA). Prior to the measurements, the sample was outgassed for 10 h under vacuum at 200 °C. The Brunauer-Emmet-Teller (BET) equation, at 0.05 < *P*/*P*_0_ < 0.15 was used for the calculation of the surface area. The pore volume was calculated at *P*/*P*_0_~0.95. The pore size distributions were obtained from the BJH (Barret–Joyner–Halenda) method applied to the adsorption branch. For morphological determination of the developed material, SEM-EDS (Scanning Electron Microscopy-Energy Dispersive X-ray Spectroscopy) was performed with JSM-6490 (JEOL, Tokyo, Japan).The HRTEM (High Resolution Transmission Electron Microscopy) and TEM-SAED (Transmission Electron Microscopy-Selected Area Electron Diffraction) were conducted with JEM 2100F (JEOL, Tokyo, Japan) operated at 200 kV. The thermal stability and quantification of the Mn adsorbed on the carbon template were determined by the TGA (Thermal gravimetric analysis) performed by Netzch STA 449C (Jupiter Ahlden, Germany). Briefly, the material was heated at 20 °C/min from 30 to 500 °C. The postmortem characterizations were performed after washing the used cathode with TEGDME and drying it in vacuum at room temperature.

### 2.4. Preparation of MOHNs/KB Cathode and Its Li–O_2_ Battery

For the preparation of the cathodes, a homogeneous ink composed of 65% MOHNs, 15% KB, and 20% PTFE binder was cast onto carbon paper discs. The discs were heated in a vacuum at 120 °C for 12 h to remove the residual solvent. The total mass loading of MOHNs/KB is 0.6 ± 0.05 mg·cm^−2^. A cathode made of KB and PTFE with the same mass loading was composed to compare the activity of the MOHNs towards the performance of the Li–O_2_ batteries.

The Li–O_2_ battery assembly was constructed within an argon-filled glovebox to avoid contaminations. Briefly, coin cells CR2025 were assembled using Li foils as the anode, Whatman glass fiber as the separator, 60 µL of 1 M LiTFSI/TEGDME as the non-aqueous electrolyte, and the coated carbon disc as the cathode. After assembling the cell, it was crimped to ensure the sealing. The coin cell batteries were placed in a closed glass chamber and flushed with pure oxygen for 1 h at room temperature prior to testing. 

### 2.5. Electrochemical Characterizations of Li–O_2_ Battery

The 2-electrode system Li–O_2_ batteries were electrochemically tested with an Electrochemical Impedance Spectroscope (EIS) (CHI 660E, Shanghai, Chiina). The Cyclic Voltammetry (CV) and Nyquist measurements were taken before the cycles. The CV was conducted at a voltage sweep rate of 1 mV/s in a voltage range of 2–4.5 V. The impedance spectrum was recorded at a perturbation amplitude of 5 mV. The galvanostatic measurements were carried out in the battery testing system (Lanhe CT 2001, Wuhan, China). The galvanostatic discharge–charge profiles (vs. Li^+^/Li) were obtained at a current density of 400 mA·g^−1^ and a restrained specific capacity of 600 mAh·g^−1^. 

## 3. Results and Discussion

### 3.1. Physicochemical Properties of MOHNs

**Figure 1:**
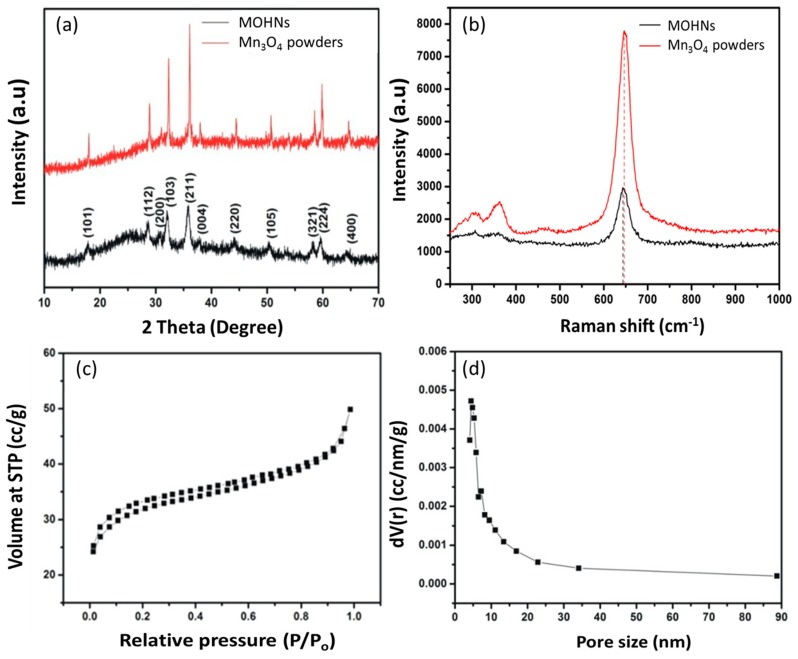
(**a**) The XRD patterns of the MOHNs and the Mn_3_O_4_ powders; (**b**) the Raman spectra of the MOHNs and the Mn_3_O_4_ powders; (**c**) the BET isotherm of the MOHNs; and (**d**) the pore size distribution of the MOHNs.

In order to compare the crystallinity of the material, a control sample of Mn_3_O_4_ was prepared by thermally annealing MnCl_2_ at 450 °C for 1 h. The XRD pattern of the MOHNs in Figure 1a is similar to the control Mn_3_O_4_ suggesting that the nanocages are the result of hausmannite type Mn_3_O_4_ (JCPDS no. 24-0734) nanocrystalline particles. The absence of any other peaks proves the phase purity of the material. The tetragonal Mn_3_O_4_ crystals belonging to the spinel class are formed of Mn^2+^ and Mn^3+^ states and can be elucidated as MnO·Mn_2_O_3_. The tetrahedral and octahedral sites of the crystals are occupied by the Mn^2+^ and Mn^3+^ ions, respectively [13,20]. This unbalance gives rise to defects that possibly prove beneficial to the catalytic activity of the cathode during ORR–OER reactions. The broadness and low intensity of the peaks in the XRD pattern of MOHNs compared to the Mn_3_O_4_ powders prove that MOHNs consist of smaller nanosized particles [21]. 

The Raman spectrum of the MOHNs in Figure 1b confirms that the characteristic peaks of Mn_3_O_4_ at 643.4, 356.7, and 306.8 cm^−1^ correspond to the A1g, T2g, and Eg active modes of Mn_3_O_4_, accordingly. The strong peak at 643.4 cm^−1^ can be attributed to the stretching vibration of the Mn–O bond in the MnO_6_ octahedral units that compose the structure of the manganese oxide. It also confirms the spinel structure of the MOHNs that corroborates with the XRD analysis [22]. The deformation vibrations of the Mn–O–Mn chains give rise to the broad band at 356.7 cm^−1^. The small peak at 306.8 cm^−1^ is primarily due to the phonon interactions with the nanoparticles [23]. There is a blue shift and broadening of the peaks of MOHNs compared to the Mn_3_O_4_ powders. This is attributed to the smaller nanosized crystals of MOHNs with large number of point defects which cause a variation in the phonon scattering [24].

The MOHNs synthesized in this work are unique in terms of their high surface area, which is difficult to attain with the spinel structure of Mn_3_O_4_. According to the IUPAC nomenclature, the nitrogen physisorption isotherm of MOHNs in Figure 1c is Type IV, indicating a loose mesoporous structure [25]. The surface area is estimated to be 90.65 m^2^·g^−1^. The pore size distribution in Figure 1d shows that it is primarily centered at a 5 nm pore diameter, which arises due to the pores between the Mn_3_O_4_ nanoparticles. The pore volume is computed to be 0.077 cc/g. The high surface area is responsible for the high concentration of exposed active centers on the surface of the material. Also, the porous cage-like structure allows faster mass transport of Li^+^ and oxygen through the internal mesoscopic pores of the materials and improves the catalytic activity [26]. 

**Figure 2:**
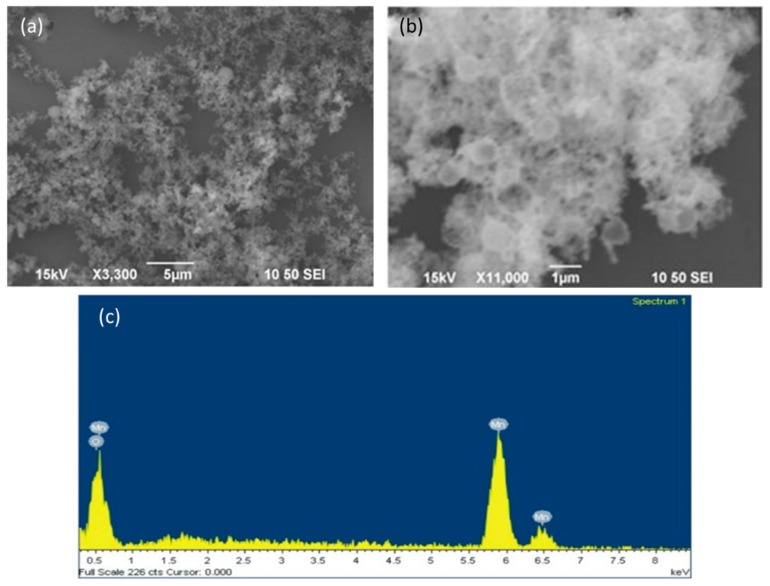
(**a**,**b**) the SEM images of the MOHNs; (**c**) the EDS spectrum of MOHNs.

**Figure 3:**
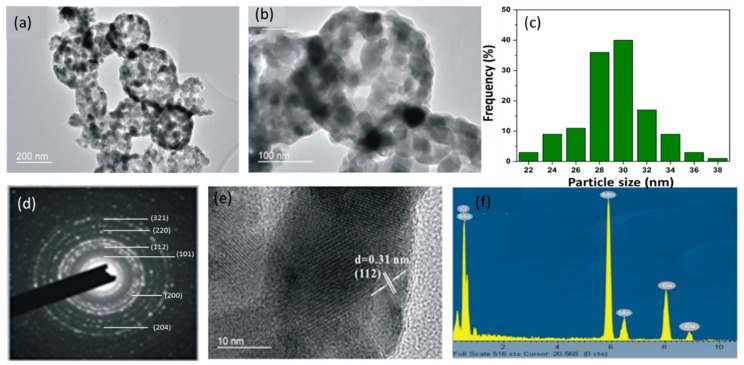
(**a**,**b**) the TEM images of the MOHNs; (**c**) the particle size distribution of Mn_3_O_4_ nanoparticles on the hollow nanocages of MOHNs; (**d**) the SAED pattern of the MOHNs; (**e**) the HRTEM image of the lattice fringe of the MOHNs; and (**f**) the EDS spectrum of MOHNs.

The SEM images of the carbon templates in Figures S1a,b show that they are smooth nanospheres typically with a diameter of 250–350 nm. It can be deduced from Figure S1c and the EDS analysis in Figure S1d that the Mn species are adsorbed on the carbon templates instead of being grafted on them. The Mn^2+^ to Cl^−^ atomic ratio is found to be greater than 3:1. Figure 2a–c reveal that the thermal treatment of the Mn adsorbed carbon templates gives rise to Mn_3_O_4_ hollow nanocages. By further probing into the morphology with TEM, it is observed from Figure 3a–c that the Mn_3_O_4_ nanoparticles of 25–35 nm aggregate on the shell of the carbon nanosphere template (~300 nm diameter) and form hollow nanocages of diameter ~270 nm. The bright diffraction rings in the SAED pattern of MOHNs in Figure 3d,e indicate the polycrystallinity of the material. The crystal planes of (112) of the diffraction ring corroborates with XRD and Raman analyses that suggest the formation of the spinel structure in Mn_3_O_4_ nanoparticles. The presence of Mn and O is further confirmed from the EDS spectrum in Figure 3f.

In order to quantify the amount of Mn adsorbed on the carbon templates, the Mn adsorbed carbon nanospheres are subjected to TGA in the air atmosphere. It is seen from Figure S2a that there is a slight mass loss at temperature <400 °C. This occurs due to loss of water molecules present in the pores of the MOHNs. A further rise in temperature causes a sharp weight loss at 445 °C and the final weight of the sample is 0.21%. According to the peak at 445 °C from the differential scanning calorimetry (DSC) plot in Figure S2b, this reaction is exothermic in nature. That also marks the point of complete oxidation and removal of the carbon template.

### 3.2. Proposed Formation Mechanism of MOHNs

**Scheme 1:**
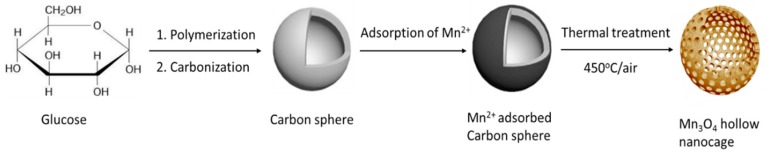
The proposed formation mechanism of MOHNs.

The proposed mechanism of the formation of the MOHNs is schematically illustrated in Scheme 1. The carbon nanospheres derived from glucose possess hydrophilic surfaces with an abundance of hydroxyls (OH) and carbonyl (C=O) groups [19,22]. In presence of the MnCl_2_ solution, these oxygen-rich species initiate the adsorption of Mn^2+^ from the solution. The SEM–EDS shows that the atomic ratio of Mn^2+^ to Cl^−^ is 3:1 while in a bulk solution during synthesis, it is calculated to be 1:2. This discrepancy is explained by the fact that the hydroxyl groups of the carbon spheres form a complex chelate with Mn^2+^ during adsorption. It prevents the metal ions from heterogeneous coagulation or being washed off [17]. This leads to the formation of an in-situ carbon sphere@metal ion core-shell composite. On thermal treatment, the metal ions undergo densification and crosslinking. The formation is explained by the chemical reactions below:

Mn^2+^ + 2OH^−^ → Mn(OH)_2_ (1)

Mn(OH)_2_ → MnO + H_2_O (2)

3MnO + 0.5O_2_ → Mn_3_O_4_ (3)

Since no alkaline medium is added, it can be safely assumed that water acts as the weak reducing agent and converts the Mn^2+^ ions to Mn(OH)_2_. It undergoes decomposition into MnO and oxidizes to Mn_3_O_4_ during thermal annealing in the presence of air [27]. Eventually, after the removal of the hard template, hollow nanocages are obtained as a result of the aggregation of metal oxide nanoparticles. Here, it is pertinent to mention that the step of ultrasonication assisted in the improvement of crystallinity of the MOHNs. Apart from facilitating the dissolution of the MnCl_2_ precursor, it also allowed a slow and uniform nucleation of the Mn_3_O_4_ nanocrystals on the surface of the carbon template. In contrast, the Mn_3_O_4_ synthesized with the stirring process cause the Mn precursor to oxidize directly, resulting in a poor and non-uniform crystal growth [21]. 

The advantages of the template process are further evaluated by synthesizing Mn_3_O_4_ by other prescribed procedures [20,28]. It should be noted from Table S1 that these reported methods are complex, energy-expensive and show the considerably low surface area as compared to the process demonstrated in this work. 

### 3.3. Electrochemical Performance of Li–O_2_ Battery

**Figure 4:**
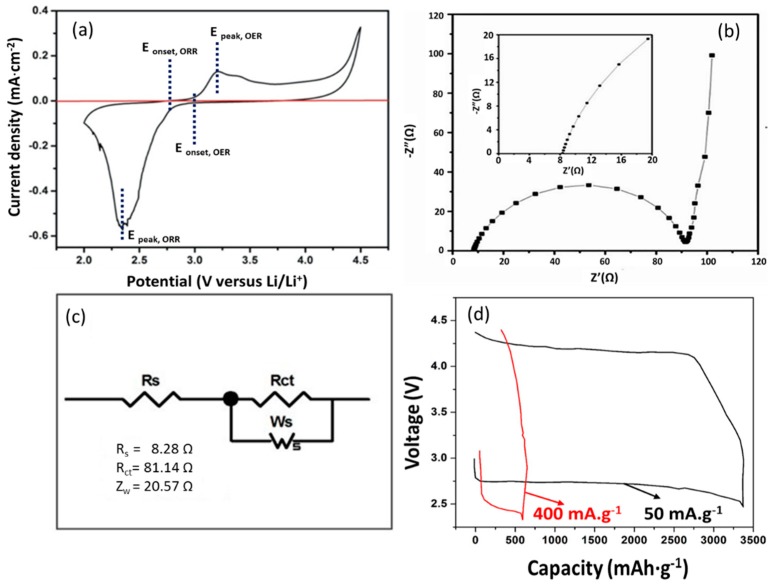
(**a**) The CV curve of MOHNs/KB cathode-based Li–O_2_ battery with LiTFSI/TEGDME as the electrolyte; (**b**) the electrochemical impedance spectrum (EIS) of the MOHNs/KB cathode-based Li–O_2_ battery. The inset is the enlarged portion of the EIS spectrum of the MOHNs/KB cathode-based Li–O_2_ battery in the range of 0–20 Ω; (**c**) the equivalent circuit model of the impedance spectrum of a MOHNs/KB cathode-based Li–O_2_ battery, and (**d**) the first discharge–charge curves of the MOHNs/KB cathode-based Li–O_2_ battery with the voltage between 2.4 and 4.4 V at a current density of 50 and 400 mA·g^−1^.

The electrocatalytic performance of the MOHNs towards ORR–OER is evaluated by using them as cathodes for nonaqueous electrolytic Li–O_2_ batteries. A nonaqueous ether-based electrolyte (TEGDME) was used because of its high stability and chemical inertness towards superoxide radicals. This, in turn, prevents the formation of lithium carbonate that block the surface of the electrodes. Further, it has been confirmed by Ahn et al. that the TEGDME is expected to undergo decomposition only above a voltage of 4.7 V. Considering the issues of electrolyte decomposition, a maximum voltage of 4.5 V was applied in this work [29,30]. It is observed from the CV curve in Figure 4a that in the presence of oxygen, the ORR onset potential of the MOHNs/KB cathode is at 2.8 V vs. Li/Li^+^. The prominent peaks of current density at 3.2 V and 2.3 V indicate the ORR and OER catalytic activity of the MOHNs, respectively [31]. The higher discharge potential of the MOHNs/KB cathode at 3.2 V than the Vulcan rubber further proves their better catalytic activity [16]. The large surface area of the MOHNs with dispersed nanoparticles are responsible for the improvement in the ORR–OER catalysis. The Nyquist plot of MOHNs/KB cathode in Figure 4b is recorded in the frequency range of 100 mHz–200 kHz. Fitting the impedance spectrum in Zview software, we obtain an equivalent circuit as in Figure 4c. The equivalent circuit model of the electrochemical process in the MOHNs/KB cathode-based Li–O_2_ battery indicates the values of *R_s_*, *R_ct_*, and *W_s_*. Here, *R_s_* indicates the ionic resistance of the electrolyte, *R_ct_* is ascribed to the charge transfer process at the anode/electrolyte and the electrolyte/cathode interface. It is clear from the parameters that the main resistance comes from the *R_ct_*. According to Younesi et al. and Assary et al., this is related to the anode/electrolyte charge transfer resistance. This resistance is expected to increase with the number of cycles due to the deposition of dendrites on the anode or the formation of a solid electrolyte interface (SEI) [32,33]. Kichambare et al. further showed that the charge transfer resistance also increases at the electrolyte/cathode interface due to increased deposition of the discharged products within the pores of the cathode [34]. The oxygen reduction reaction taking place during discharge induces the formation of LiO_2_ as described in Equations (4) and (5). The resistance, however, reduces during the discharge due to the dominance of the oxidation process that takes place at higher voltages with the progress of the charging process, following the decomposition of the build-up discharged products. The reaction Li_2_O_2_ → 2Li^+^ + O_2_ + 2e^−^ occurs during the charging process. The nearly linear slope of Warburg impedance *W_s_* provides an indication of the high diffusivity of the Li^+^ ions through the MOHNs/KB cathode due to its porous structure [35,36,37]. Further investigations on the impedance of the MOHNs/KB cathode-based Li–O_2_ battery will be conducted to understand the electrocatalytic process. It is observed in Figure 4d that at a current density of 50 mA·g^−1^, the MOHNs/KB cathode-based Li–O_2_ battery exhibits a flat discharge voltage plateau and the corresponding specific discharge capacity is estimated to be as high as 3380 mAh·g^−1^ (~2.03 mAh·cm^−2^). However, at a higher current density of 400 mA·g^−1^, the same cathode-based Li–O_2_ battery show a slant discharge voltage plateau and an output of only 593 mAh·g^−1^ (~0.35 mAh·cm^−2^) discharge capacity. The poor performance of the MOHNs/KB cathode-based Li–O_2_ battery at high current densities can be attributed to two possibilities. Firstly, at higher discharge rates, the gradient of the oxygen concentration across the cathode is elevated. This leads to restriction of the reaction regime at a close proximity to the gas-cathode interface [38]. Secondly, it is more difficult to remove the build-up discharged products such as lithium peroxide at the higher current density of 400 mA·g^−1^ [39,40]. 

**Figure 5:**
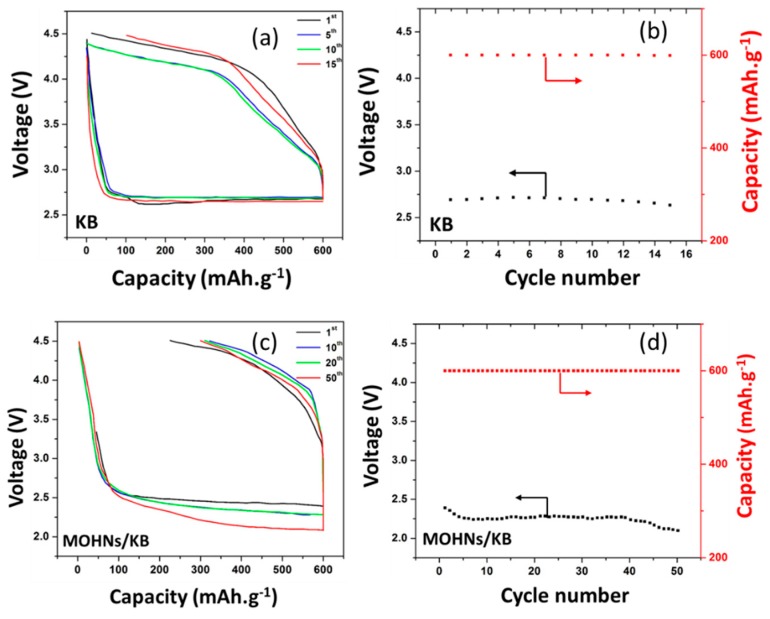
(**a**) The discharge–charge curves and (**b**) the cyclic stability of the KB cathode-based Li–O_2_ battery; (**c**) the discharge–charge curves and (**d**) the cyclic stability of the MOHNs/KB cathode-based Li–O_2_ battery. All tests of Li–O_2_ batteries were subjected to a restrained specific capacity of 600 mAh·g^−1^ and a specific current of 400 mA·g^−1^.

The galvanostatic discharge–charge profiles of the Li–O_2_ batteries with a KB cathode and a MOHNs/KB cathode are depicted in Figure 5a,b. The discharge voltage in both cases decreases till they reach a steady-state value. The difference between the open circuit voltage and steady-state voltage for the first discharge cycle, ∆*E*_discharge_ is 0.94 and 1.76 V for the MOHNs/KB and KB cathode-based Li–O_2_ batteries, respectively. The reduced overpotential is the result of the catalytic activity of the MOHNs [18,41]. Therefore, it can be safely concluded that the performance of the Li–O_2_ batteries depend heavily on the properties of the cathode. 

In evaluating the cycling performance of the Li–O_2_ batteries, the discharge capacity is limited to 600 mAh·g^−1^ to avoid the decomposition of the electrolyte [42]. It is shown in Figure 5c,d that the MOHNs/KB cathode-based Li–O_2_ battery can sustain 50 discharge–charge cycles while delivering a stable reversible capacity of 600 mAh·g^−1^ throughout. The discharge cut-off voltage for MOHNs/KB cathode-based Li–O_2_ battery is about 2–2.4 V. In contrast, the KB cathode-based Li–O_2_ battery has a higher discharge cut-off voltage at 2.6–2.7 V and starts showing signs of deterioration only after 15 cycles. The high diffusion of oxygen through the nanocages of the MOHNs improve the catalytic activity but the fast reaction rate also accounts for the side reactions. It is proposed that the low recharge efficiency of the MOHNs cathode is due to the presence of side reactions and passivation of the electrodes [43,44,45]. The MOHNs/KB cathode outperforms the α-MnO_2_ nanowires, FeCo_2_O_4_ or RuO_2_ foam cathodes when used in Li–O_2_ batteries [10,42,46]. This is attributed to the mesoporous structure of the Mn_3_O_4_ crystals aggregated to form the 250 nm diameter nanocages which facilitate the diffusion of a large amount of oxygen and Li ions that escalate the ORR–OER activity. The cyclic stability is maintained because the Li_2_O_2_ produced as the side product gets decomposed and is unable to block the exceptionally large pores of the MOHNs. After 50 or more cycles, the discharged products become more difficult to be decomposed by the passivated active sites and even the large pores of MOHNs cannot accommodate the high amount of discharge products. Hence, the capacity drops and the battery reaches its limit [47,48,49]. 

It is proposed that point defects and oxygen vacancies of MOHNs are responsible for the enhanced catalytic activity of MOHNs/KB cathode-based Li–O_2_ battery. During heating to 450 °C, the average oxidation state in Mn_3_O_4_ is less than the stoichiometric state of 4 [50]. These oxygen vacancies improve the electronic conductivity of the MOHNs and facilitate better accessibility of Li^+^ and O_2_^−^ and the decomposition of Li_2_O_2_. On the other hand, the porous structure improves the oxygen diffusion through the electrode-electrolyte interfaces [51,52,53].

**Figure 6:**
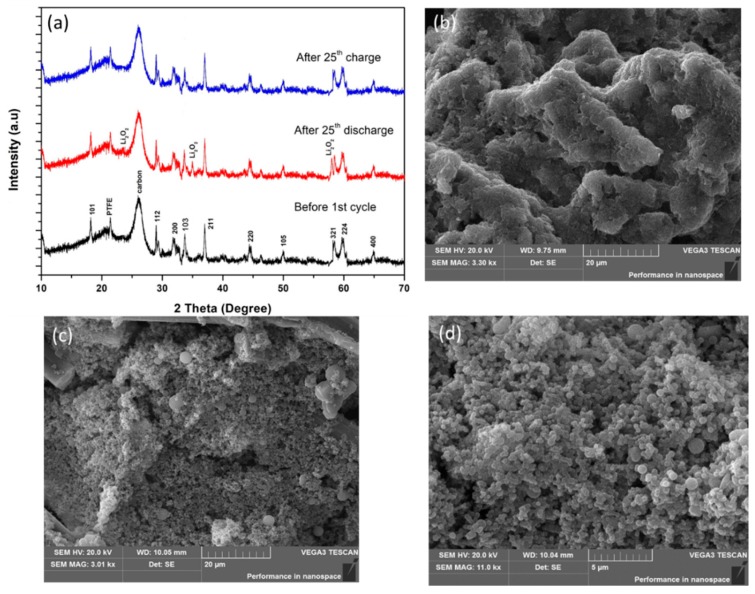
(**a**) The XRD patterns of the MOHNs/KB cathode before the cycle, after the 25th discharge cycle, and after the 25th recharge cycle; (**b**) the SEM image of the MOHNs/KB cathode after the 25th discharge; (**c**,**d**) the SEM image of the MOHNs/KB cathode after the 25th recharge.

The postmortem XRD patterns of the MOHNs/KB cathode of the Li–O_2_ battery in Figure 6a show that the pristine MOHNs/KB cathode before cycling has prominent peaks of PTFE and carbon from KB (Figure S3), which were used in the cathode preparation as a binder and conductive carbon, respectively. After the 25th discharge cycle, the MOHNs/KB cathode showed the presence of Li_2_O_2_. This development of side products was also confirmed by the SEM images. Figure 6b depicts the formation of amorphous Li_2_O_2_ instead of well-defined toroids on the surface of the MOHNs/KB cathode after the 25th discharge cycle. However, due to the catalytic activity of the porous MOHNs, the decomposition of Li_2_O_2_ occurs after the 25th charge cycle. Hence, the cathode surface assumes a morphology similar to its pristine form again. This reversibility of performance can be easily verified from the comparison of the SEM images of pristine and postmortem MOHNs shown in Figure 2a,b and Figure 6c,d, respectively. The argument is also corroborated by the XRD pattern of the MOHNs/KB cathode after the 25th cycle of charging, which shows the absence of Li_2_O_2_. The absence of any peaks of Li_2_CO_3_ proves the high stability and absence of decomposition of the ether-based electrolyte under the applied voltage range [40].

Following the electrocatalytic processes analyzed from the equivalent circuit in Figure 4c and the discharged products formation of Li_2_O_2_ verified with SEM image in Figure 6b, we hypothesize that the non-crystalline amorphous Li_2_O_2_ deposition on the MOHNs/KB cathodes takes place by surface-mediated growth as proposed by Nazar et al. A high concentration of lithium superoxide is generated on the surface during the discharge of the MOHNs/KB cathode-based Li–O_2_ battery. These species undergo disproportionation via surface migration and results in the nucleation of poorly shaped low crystalline Li_2_O_2_. The process of formation of surface-mediated amorphous Li_2_O_2_ during the discharge of the MOHNs/KB cathode-based Li–O_2_ battery is described in Equations (4) and (5), where LiO_2_ and Li_2_O_2_ are surface adsorbed species [40].

O_2_ + Li^+^ + e^−^ → LiO_2_ (4)

LiO_2_ + Li^+^ + e^−^ → Li_2_O_2_ (5)

## 4. Conclusions

In summary, we report a facile strategy to produce non-precious spinel-type manganese oxide (Mn_3_O_4_) with a high surface area of 90.65 m^2^·g^−1^ and defects in the form of oxygen vacancies. The unique nanocage structure with a cage diameter of 250 nm facilitates the easy diffusion of the Li-ions and the inter-particle pores provide a short diffusion pathway for the reactants and products for better electrocatalytic activity. Therefore, the MOHNs/KB cathode-based Li–O_2_ batteries exhibit cyclic stability for a minimum of 50 cycles at a current density of 400 mA·g^−1^. The postmortem characterizations also provide evidence to the successful decomposition of Li_2_O_2_ during recharge due to the high catalytic activity and low resistivity of the MOHNs/KB cathodes. Such high performance provides the promise of synthesizing the hybrid spinel metal oxides with Co, Fe, and Pd as the next-generation cathodes for Li–O_2_ batteries as well as for Zn–air and Mg–air batteries, which are promising technologies for electric vehicles. The high capacity of 3380 mAh·g^−1^ (~2.03 mAh·cm^−2^) achieved by the MOHNs/KB cathode-based Li–O_2_ battery paves the way to popularize the template synthesis of high surface area metal oxides as cathodes. Eventually, this work shows promising streaks to use cheap transition metal oxides as cathodes for Li–O_2_ batteries while eliminating the use of expensive platinum, graphene, and carbon nanotubes.

## Supplementary Materials

The following are available online at http://www.mdpi.com/2079-4991/8/5/308/s1, Figure S1. (a) SEM image of carbon spheres; (b) TEM image of carbon spheres; (c) TEM image and (d) EDS of Mn-adsorbed carbon spheres, Figure S2. (a) TGA and (b) DSC plots of Mn-adsorbed carbon spheres, Table S1. Comparison of synthesis procedures and surface area of Mn_3_O_4_ hollow structures, Figure S3. XRD pattern of KB carbon.

## References

[1] Larcher D., Tarascon J.-M. (2014). Towards greener and more sustainable batteries for electrical energy storage. Nat. Chem..

[2] Bruce P.G., Freunberger S.A., Hardwick L.J., Tarascon J.-M. (2011). Li–O_2_ and Li–S batteries with high energy storage. Nat. Mater..

[3] Eftekhari A., Ramanujam B. (2017). In pursuit of catalytic cathodes for lithium–oxygen batteries. J. Mater. Chem. A.

[4] Shao Y., Park S., Xiao J., Zhang J.G., Wang Y., Liu J. (2012). Electrocatalysts for nonaqueous lithium-air batteries: Status, challenges, and perspective. ACS Catal..

[5] Beattie S.D., Manolescu D.M., Blair S.L. (2009). High-capacity lithium–air cathodes. J. Electrochem. Soc..

[6] Imanishi N., Yamamoto O. (2014). Rechargeable lithium-air batteries: Characteristics and prospects. Mater. Today.

[7] Guo X., Sun B., Su D., Liu X., Liu H., Wang Y., Wang G. (2017). Recent developments of aprotic lithium-oxygen batteries: Functional materials determine the electrochemical performance. Sci. Bull..

[8] Ma Z., Shao G., Fan Y., Wang G., Song J., Shen D. (2016). Construction of hierarchical α-MnO_2_ nanowires@ultrathin δ-MnO_2_ nanosheets core-shell nanostructure with excellent cycling stability for high-power asymmetric supercapacitor electrodes. ACS Appl. Mater. Interfaces.

[9] Park M.-S., Kim J.-H., Kim K.J., Jeong G., Kim Y.-J. (2013). Morphological modification of α-MnO_2_ catalyst for use in Li/air batteries. J. Nanosci. Nanotechnol..

[10] Débart A., Paterson A.J., Bao J., Bruce P.G. (2008). α-MnO_2_ nanowires: A catalyst for the O_2_ electrode in rechargeable lithium batteries. Angew. Chem. Int. Ed..

[11] Liu B., Sun Y., Liu L., Xu S., Yan X. (2018). Advances in manganese-based oxides cathodic electrocatalysts for Li-air batteries. Adv. Funct. Mater..

[12] Park M.-S., Kim J., Kim K.J., Lee J.-W., Kim J.H., Yamauchi Y. (2015). Porous nanoarchitectures of spinel-type transition metal oxides for electrochemical energy storage systems. Phys. Chem. Chem. Phys..

[13] Pal S., Lal S. (2017). Orbital and spin ordering physics of the Mn_3_O_4_ spinel. Phys. Rev. B.

[14] Lv K., Zhang Y., Zhang D., Ren W., Sun L. (2017). Mn_3_O_4_ nanoparticles embedded in 3D reduced graphene oxide network as anode for high-performance lithium ion batteries. J. Mater. Sci. Mater. Electron..

[15] Ramírez A., Hillebrand P., Stellmach D., May M.M., Bogdanoff P., Fiechter S. (2014). Evaluation of MnOx, Mn_2_O_3_, and Mn_3_O_4_ electrodeposited films for the oxygen evolution reaction of water. J. Phys. Chem. C.

[16] Cao Y., Or S.W. (2016). Enhanced cyclability in rechargeable Li–O_2_ batteries based on Mn_3_O_4_ hollow nanocage/Ketjenblack catalytic air cathode. IEEE Trans. Mag..

[17] Li Q., Xu P., Zhang B., Tsai H., Wang J., Wang H.-L., Wu G. (2013). One-step synthesis of Mn_3_O_4_/reduced graphene oxide nanocomposites for oxygen reduction in nonaqueous Li–O_2_ batteries. Chem. Commun..

[18] Jung K.-N., Lee J.-I., Yoon S., Yeon S.-H., Chang W., Shin K.-H., Lee J.-W. (2012). Manganese oxide/carbon composite nanofibers: Electrospinning preparation and application as a bi-functional cathode for rechargeable lithium–oxygen batteries. J. Mater. Chem..

[19] Abdelaal H.M. (2015). Template-assisted synthesis of metal oxide hollow spheres utilizing glucose derived-carbonaceous spheres as sacrificial templates. J. Adv. Chem. Eng..

[20] Yue J., Gu X., Chen L., Wang N., Jiang X., Xu H., Yang J., Qian Y. (2014). General synthesis of hollow MnO_2_, Mn_3_O_4_ and MnO nanospheres as superior anode materials for lithium ion batteries. J. Mater. Chem. A.

[21] Lei S., Tang K., Fang Z., Zheng H. (2006). Ultrasonic-assisted synthesis of colloidal Mn_3_O_4_ nanoparticles at normal temperature and pressure. Cryst. Growth Des..

[22] Yang L.X., Liang Y., Chen H., Meng Y.F., Jiang W. (2009). Controlled synthesis of Mn_3_O_4_and MnCO_3_ in a solvothermal system. Mater. Res. Bull..

[23] Toufiq A.M., Wang F., Javed U.-Q.-A., Li Q., Li Y., Khan M. (2014). Synthesis, characterization and photoluminescent properties of 3D nanostructures self-assembled with Mn_3_O_4_ nanoparticles. Mater. Express.

[24] Zuo J., Xu C., Liu Y., Qian Y. (1998). Crystallite size effects on the Raman spectra of Mn_3_O_4_. Nanostruct. Mater..

[25] Guo H., Li T., Chen W., Liu L., Qiao J., Zhang J. (2015). Self-assembly formation of hollow Ni-Fe-O nanocage architectures by metal-organic frameworks with high-performance lithium storage. Sci. Rep..

[26] Dutta A., Gupta D., Patra A.K., Saha B., Bhaumik A. (2014). Synthesis of 5-hydroxymethylfurural from carbohydrates using large-pore mesoporous tin phosphate. ChemSusChem.

[27] Dhaouadi H., Ghodbane O., Hosni F., Touati F. (2012). Nanoparticles: Synthesis, characterization, and dielectric Properties. ISRN Spectrosc..

[28] Fang M., Tan X., Liu M., Kang S., Hu X., Zhang L. (2011). Low-temperature synthesis of Mn_3_O_4_ hollow-tetrakaidecahedrons and their application in electrochemical capacitors. CrystEngComm.

[29] Ahn S.M., Suk J., Kim D.Y., Kang Y., Kim H.K., Kim D.W. (2017). High-performance lithium-oxygen battery electrolyte derived from optimum combination of solvent and lithium salt. Adv. Sci..

[30] Kim B.G., Kim H.J., Back S., Nam K.W., Jung Y., Han Y.K., Choi J.W. (2015). Improved reversibility in lithium-oxygen battery: Understanding elementary reactions and surface charge engineering of metal alloy catalyst. Sci. Rep..

[31] Augustin M., Fenske D., Parisi J. (2016). Study on electrolyte stability and oxygen reduction reaction mechanisms in the presence of manganese oxide catalysts for aprotic lithium–oxygen batteries. Energy Technol..

[32] Younesi R., Hahlin M., Roberts M., Edström K. (2013). The SEI layer formed on lithium metal in the presence of oxygen: A seldom considered component in the development of the Li–O_2_ battery. J. Power Sources.

[33] Assary R.S., Lu J., Du P., Luo X., Zhang X., Ren Y. (2013). The effect of oxygen crossover on the anode of a Li–O_2_ battery using an ether-based solvent: Insights from experimental and computational studies. ChemSusChem.

[34] Kichambare P., Kumar J., Rodrigues S., Kumar B. (2011). Electrochemical performance of highly mesoporous nitrogen doped carbon cathode in lithium–oxygen batteries. J. Power Sources.

[35] Mirzaeian M., Hall P.J. (2010). Characterizing capacity loss of lithium oxygen batteries by impedance spectroscopy. J. Power Sources.

[36] Zhang B., Liu Y., Huang Z., Oh S., Yu Y., Mai Y.-W., Kim J.-K. (2012). Urchin-like Li_4_Ti_5_O_12_–carbon nanofiber composites for high rate performance anodes in Li-ion batteries. J. Mater. Chem..

[37] Landa-Medrano I., de Larramendi I.R., Ortiz-Vitoriano N., Pinedo R., de Larramendi J.I., Rojo T. (2014). In situ monitoring of discharge/charge processes in Li–O_2_ batteries by electrochemical impedance spectroscopy. J. Power Sources.

[38] Read J. (2002). Characterization of the lithium/oxygen organic electrolyte battery. J. Electrochem. Soc..

[39] Wang F., Xu Y.H., Luo Z.K., Pang Y., Wu Q.X., Liang C.S., Chen J., Liu D., Zhang X.H. (2014). A dual pore carbon aerogel based air cathode for a highly rechargeable lithium-air battery. J. Power Sources.

[40] Adams B.D., Radtke C., Black R., Trudeau M.L., Zaghib K., Nazar L.F. (2013). Current density dependence of peroxide formation in the Li–O_2_ battery and its effect on charge. Energy Environ. Sci..

[41] Kwak W.-J., Lau K.C., Shin C.-D., Amine K., Curtiss L.A., Sun Y.-K. (2015). A Mo_2_C/Carbon nanotube composite cathode for lithium–oxygen batteries with high energy efficiency and long cycle life. ACS Nano.

[42] Kwak K.-H., Kim D.W., Kang Y., Suk J. (2016). Hierarchical Ru- and RuO_2_-foams as high performance electrocatalysts for rechargeable lithium–oxygen batteries. J. Mater. Chem. A.

[43] Lu Y.-C., Gallant B.M., Kwabi D.G., Harding J.R., Mitchell R.R., Whittingham M.S., Shao-Horn Y. (2013). Lithium–oxygen batteries: Bridging mechanistic understanding and battery performance. Energy Environ. Sci..

[44] Yin W., Shen Y., Zou F., Hu X., Chi B., Huang Y. (2015). Metal-organic framework derived ZnO/ZnFe_2_O_4_/C nanocages as stable cathode material for reversible lithium-oxygen batteries. ACS Appl. Mater. Interfaces.

[45] Ren X., Zhang S.S., Tran D.T., Read J. (2011). Oxygen reduction reaction catalyst on lithium/air battery discharge performance. J. Mater. Chem..

[46] Mohamed S.G., Tsai Y.-Q., Chen C.-J., Tsai Y.-T., Hung T.-F., Chang W.-S., Liu R.-S. (2015). Ternary spinel MCo_2_O_4_ (M = Mn, Fe, Ni, and Zn) porous nanorods as bifunctional cathode materials for lithium–O_2_ batteries. ACS Appl. Mater. Interfaces.

[47] Lu Y.-C., Gasteiger H.A., Parent M.C., Chiloyan V., Shao-Horn Y. (2010). The influence of catalysts on discharge and charge voltages of rechargeable Li–oxygen batteries. Electrochem. Solid-State Lett..

[48] Tan P., Wei Z.H., Shyy W., Zhao T.S., Zhu X.B. (2016). A nano-structured RuO_2_/NiO cathode enables the operation of non-aqueous lithium–air batteries in ambient air. Energy Environ. Sci..

[49] Chen Y.M., Xie A., Zhu Y. (2015). Cyclability of a lithium-oxygen battery containing *N*-Methyl-2-Pyrrolidone and a vertically aligned carbon nanotube cathode. ChemElectroChem.

[50] Mette K., Bergmann A., Tessonnier J.-P., Hävecker M., Yao L., Ressler T., Schlögl R., Strasser P., Behrens M. (2012). Nanostructured manganese oxide supported on carbon nanotubes for electrocatalytic water splitting. ChemCatChem.

[51] Risch M., Stoerzinger K.A., Han B., Regier T.Z., Peak D., Sayed S.Y., Wei C., Xu Z., Shao-Horn Y. (2017). Redox processes of manganese oxide in catalyzing oxygen evolution and reduction: An in situ soft X-ray absorption spectroscopy study. J. Phys. Chem. C.

[52] Recham N., Chotard J.N., Dupont L., Delacourt C., Walker W., Armand M., Tarascon J.M. (2010). A 3.6 v lithium-based fluorosulphate insertion positive electrode for lithium-ion batteries. Nat. Mater..

[53] Li L., Feng X., Nie Y., Chen S., Shi F., Xiong K., Ding W., Qi X., Hu J., Wei Z. (2015). Insight into the effect of oxygen vacancy concentration on the catalytic performance of MnO_2_. ACS Catal..

