# Correction: Chatterjee, A.; et al. Transition Metal Hollow Nanocages as Promising Cathodes for the Long-Term Cyclability of Li–O_2_ Batteries. *Nanomaterials* 2018, *8*, 308

**DOI:** 10.3390/nano8100748

**Published:** 2018-09-20

**Authors:** Amrita Chatterjee, Siu Wing Or, Yulin Cao

**Affiliations:** 1 Department of Electrical Engineering, The Hong Kong Polytechnic University, Hung Hom, Kowloon, Hong Kong; amrita.chatterjee@polyu.edu.hk (A.C.); caoyulin@szpt.edu.cn (Y.C.); 2 Physics Laboratory, Industrial Training Center, Shenzhen Polytechnic, Shenzhen 518055, China

The authors wish to add the following information to this paper [1].

The last paragraph of Section 1 in the Introduction has been replaced by the following two paragraphs:

One of the drawbacks of using these spinel structured oxides is their low surface area [12]. In our previous work [16], we have announced the preliminary results and initial observations on the basic morphology and magnetism of a highly porous spinel-type, Mn_3_O_4_, called Mn_3_O_4_ hollow nanocages (MOHNs), in addition to the general electrochemical performance of MOHNs/Ketjenblack (KB) cathode-based Li−O_2_ batteries. It has been demonstrated that the use of a simple facile template assisted growth technique is capable of producing crystalline paramagnetic MOHNs composed of many 25 nm mean diameter Mn_3_O_4_ nanoparticles, loosely agglomerated together to form the shell of a mesoporous hollow nanocage structure with a large mean diameter of 250 nm and a high surface area of 90.65 m^2^·g^−1^. Moreover, the resulting MOHNs/KB cathode-based Li−O_2_ batteries exhibit more than 50 discharge–charge cycles at a reversible restrained specific capacity of 600 mAh·g^−1^ and a specific current of 400 mA·g^−1^.

This paper is extended from the previous proceedings paper [16]. It broadens the previous focus on the physical aspect of MOHNs to the physicochemical aspect of MOHNs. We thereby provide a more comprehensive evaluation and elaboration on the physicochemical properties and formation mechanism of MOHNs, as well as the electrochemical performance of MOHNs/KB cathode-based Li−O_2_ batteries. An analysis of death batteries is also performed, in order to understand how the mesoporous hollow nanocage structure of MOHNs provides a pathway for better diffusion of reactants and products, how it prevents the blockage of pores from Li_2_O_2_, and how it improves the cyclic stability of Li–O_2_ batteries.

The figure captions of Figures 2–5 are added with the following statements:

Figure 2a is reproduced with permission from [16]. Copyright IEEE, 2016.
Figure 3b is reproduced with permission from [16]. Copyright IEEE, 2016.
Figure 4a,d are reproduced with permission from [16]. Copyright IEEE, 2016.
Figure 5 is reproduced with permission from [16]. Copyright IEEE, 2016.

The authors regret any inconvenience or misunderstanding caused by these errors. The manuscript will be updated and the original will remain available on the article webpage.
