# Supplementary material for: Transition Metal Hollow Nanocages as Promising Cathodes for the Long-Term Cyclability of Li–O2 Batteries
Source: Nanomaterials (Basel). 2018 May 7;8(5):308. doi: 10.3390/nano8050308 (PMC5977322; doi:10.3390/nano8050308)
Supplement: Supplementary file 1 [file nanomaterials-08-00308-s001.pdf]

Supplementary Information

Transition Metal Hollow Nanocages as Promising Cathodes for the Long-Term Cyclability of Li–O_2 Batteries

Amrita Chatterjee [1], Siu Wing Or [1,*] and Yulin Cao [1,2]

[1] Department of Electrical Engineering, The Hong Kong Polytechnic University, Hung Hom, Kowloon, Hong Kong; amrita.chatterjee@polyu.edu.hk (A.C.); caoyulin@szpt.edu.cn (Y.C.)
[2] Physics Laboratory, Industrial Training Center, Shenzhen Polytechnic, Shenzhen 518055, China
* Correspondence: eeswor@polyu.edu.hk; Tel.: +852-34003345

Received: 19 March 2018; Accepted: 2 May 2018; Published: date

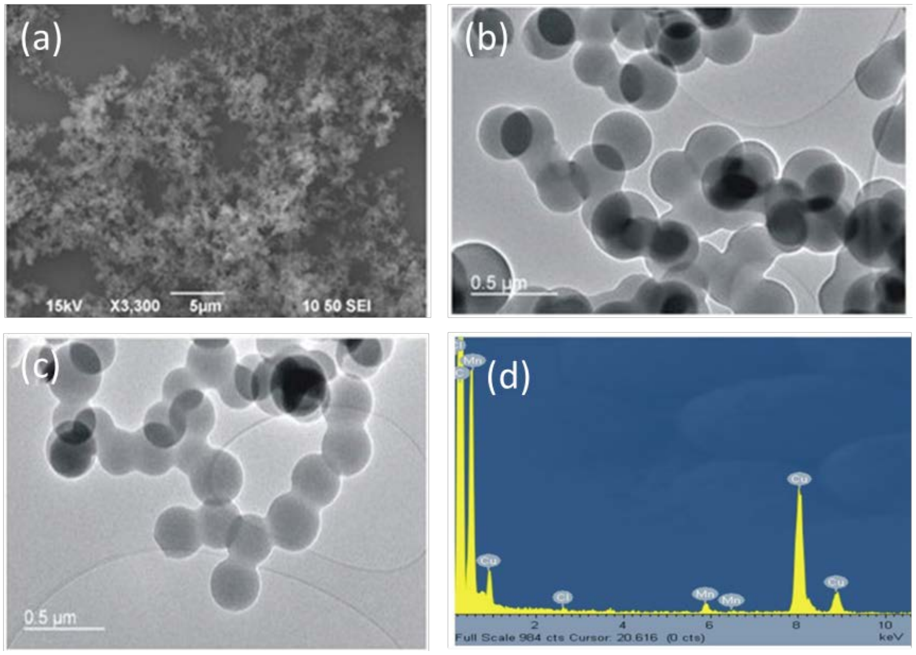

Figure S1. (a) SEM image of carbon spheres; (b) TEM image of carbon spheres; (c) TEM image and (d) EDS of Mn-adsorbed carbon spheres.

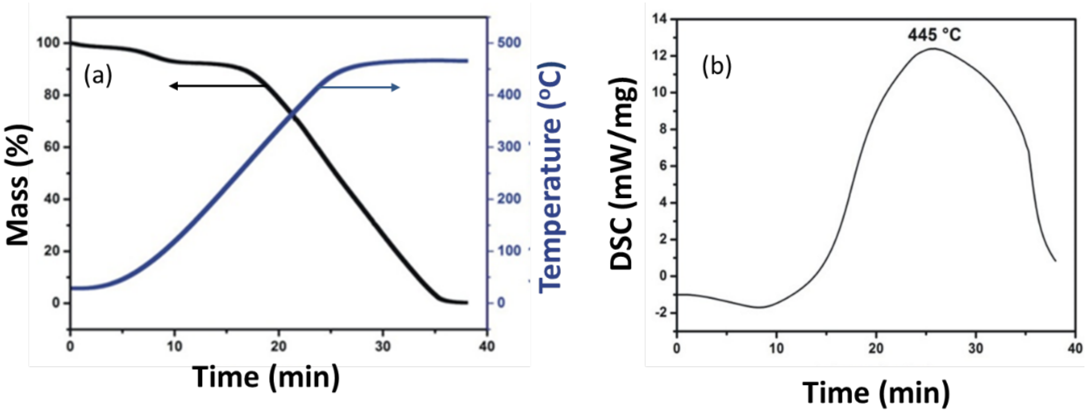

Figure S2. (a) TGA and (b) DSC plots of Mn-adsorbed carbon spheres.

Table S1. Comparison of synthesis procedures and surface area of Mn_3O_4 hollow structures

Morphology	Precursors	Preparation methods	S_{BET} ($m^2.g^{-1}$)	Ref.
Mn_3O_4 hollow spheres	$KMnO_4$	1) Carbon spheres+$KMnO_4$, hydrothermal treatment, 100 °C for 45 min;<br>2) calcination, 300 °C for 10 h in air;<br>3) annealing, 280 °C for 3 h in Ar/H2.	59	[S1]
Mn_3O_4 hollow tetrakaidecahedrons	Mn + NaClO4 + NaOH	1) Mn + $NaClO_4$ + NaOH, bubbled with pure N2;<br>2) hydrothermal treatment, 200 °C for 24 h;<br>3) calcination, 600 °C for 2h in air.	37.16	[S2]
Mn_3O_4 hollow nanocages	$MnCl_2$	Carbon spheres+$MnCl_2$, calcination, 450 °C for 1 h in air.	90.65	This work

S_{BET}: Specific surface area calculated by BET method.

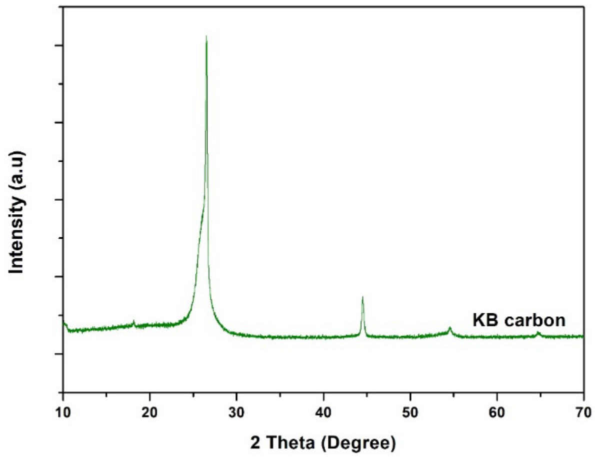

Figure S3. XRD pattern of KB carbon.

References

S1. Yue, J.; Gu, X.; Chen, L.; Wang, N.; Jiang, X.; Xu, H.; Yang, J.; Qian, Y. General synthesis of hollow MnO_2, Mn_3O_4 and MnO nanospheres as superior anode materials for lithium ion batteries. *J. Mater. Chem. A* **2014**, *2*, 17421–17426.

S2. Zhang, G. Q.; Zheng, J. P.; Liang, R.; Zhang, C.; Wang, B.; Hendrickson, M.; Plichta, E. J. Lithium–Air Batteries Using SWNT/CNF Buckypapers as Air Electrodes. *J. Electrochem. Soc.* **2010**, *157*, A953.
